# Osteocyte-vascular Interactions in Bone Physiology and Pathology

**DOI:** 10.1007/s11914-026-00978-x

**Published:** 2026-08-05

**Authors:** Léa Gellée, Delphine B. Maurel

**Affiliations:** 1 Tissue Bioengineering Laboratory (BioTis), Inserm U1026, University of Bordeaux, Bordeaux, 33000 France; 2 Pharmaceutical Sciences Department, Université de Bordeaux, Bordeaux, France

**Keywords:** Signalling molecules, Bone repair, Endothelial cell, Bone vasculature, Lymphatics, Osteocytes communication

## Abstract

**Purpose of Review:**

Through their extensive lacuno-canalicular network, osteocytes act as key regulators of bone remodelling and have more recently been recognized as contributors to bone repair. However, these processes occur in a highly vascularized tissue, and the relationship between osteocytes and bone vasculature remains poorly understood. This review summarizes current knowledge on osteocyte-vascular interactions, with a focus on communication mechanisms and their relevance to bone physiology, aging, and disease.

**Recent Findings:**

Emerging evidence indicates that osteocyte-vascular communication occurs through several modalities. Indirect signalling involves paracrine factors secreted by osteocytes, including VEGF, sclerostin, neuropeptide Y, as well as extracellular vesicles, which modulate ECs’ behaviour. Direct communication has also been suggested through physical interactions between osteocytes and blood vessels, including mitochondrial transfer and potentially gap junctions. Recent studies have also identified lymphatic vessels within bone, raising the possibility of previously unrecognized interactions between osteocytes and the lymphatic vasculature. Together, these findings highlight diverse modes of communications and associated signalling pathways, through which osteocytes may regulate vascular function within bone.

**Summary:**

Despite emerging insights into osteocyte-bone vasculature crosstalk, the underlying mechanisms remain incompletely understood. Advances in experimental tools may improve our knowledge of this bidirectional dialogue, providing new perspectives on bone physiology, skeletal pathologies, and potential therapeutic strategies targeting this interaction.

## Introduction

For many years, osteocytes were considered passive cells because of their inaccessible location within the mineralized bone matrix. Advances in imaging and experimental techniques have since established osteocytes as central regulators of skeletal homeostasis [1]. Representing 90–95% of bone cells, osteocytes form an extensive lacuno-canalicular network that enables mechanosensation and communication with neighbouring bone cells [2–4]. Through this highly interconnected network, they regulate bone remodelling by controlling osteoblast and osteoclast activity and contribute to systemic phosphate and vitamin D homeostasis through endocrine functions [3]. Emerging evidence also supports a role for osteocytes in bone repair [5–8]. Despite these advances, key aspects of osteocyte biology remain poorly understood, particularly regarding their interactions with the vascular compartment.

Bone vasculature is a highly specialized network essential for oxygen and nutrient delivery, waste removal, and skeletal development, remodelling, and regeneration. Several bone pathologies result from an alteration of the vascular network, such as the progression of certain cancers or Paget’s disease [9]. Various conditions lead to an interruption of bone vascularisation, such as a fracture or a surgical incident, inducing osteonecrosis [10, 11]. Beyond these functions, endothelial cells regulate their microenvironment through the secretion of angiocrine factors and interactions with resident bone cells [12–16]. While endothelial-osteoprogenitor communication has been extensively studied, interactions between osteocytes and the vascular compartment remain less well understood.

Several studies have reported physical interactions between blood vessels and osteocytes, suggesting the existence of direct cellular communication, but the nature of these interactions has been little studied [17–21]. Osteocyte communication has often been considered in a restrictive manner, focusing on a limited number of osteocyte factors. This view likely underestimates the complexity of osteocyte interactions within the bone microenvironment, particularly with the vascular network. Indeed, emerging work indicates that osteocytes and the vascular compartment may communicate through multiple mechanisms, including paracrine secretion and exosome-mediated pathways [22–25]. A better understanding of these interactions may provide important insights into bone physiology, disease pathogenesis, and potential therapeutic strategies.

In this review, we synthesize the current knowledge regarding osteocyte-vascular interactions, addressing the different modes of communication, the associated signalling pathways, and the contribution of these interactions to skeletal pathologies and aging.

## Bone Vasculature

### Blood Vascular Architecture in Bone

Bone tissue is highly vascularized and the architecture of the intraosseous vascular network has been investigated for many decades [26, 27]. In long bones, blood supply is provided by central, metaphyseal-epiphyseal, and periosteal arteries, whereas venous drainage involves epiphyseal, metaphyseal, and centromedullary veins. Recent light-sheet fluorescence microscopy studies have also identified transcortical vessels (TCVs), a network of arterial and venous capillaries connecting the periosteum and endosteum through cortical bone in both mice and humans [28, 29].

Blood vessels of different bone regions have distinct structures with EC heterogeneity. Bone capillary endothelial cells (ECs) are classified into two subtypes, H (“High”) and L (“Low”), based on endomucin and CD31/PECAM1 expression (Platelet EC Adhesion Molecule 1). Type H ECs, characterized by high levels of these markers, are located in the metaphysis and endosteum of long bones and are associated with osteogenesis through interactions with Osterix-expressing osteoprogenitors that couple angiogenesis and osteogenesis [13, 30]. In contrast, type L ECs form a branched vascular network in the bone marrow of the diaphysis, display low endomucin and CD31/PECAM1 expression, and are not associated with osteoprogenitors. A third subtype, termed type E ECs, has recently been identified during embryonic and early postnatal development [31].

Vascular organization in flat and short bones, such as the calvaria, has similarities with epiphyseal-metaphyseal networks and evidence suggests the presence of vessel subtypes comparable to type H capillaries [32, 33].

## Bone-blood Vasculature Crosstalk in Osteogenesis and Bone Repair

This blood vascular network is essential not only for oxygen and nutrient delivery but also for bone formation, maintenance, and repair. Angio-osteogenic coupling is a tightly regulated interaction between ECs and osteogenic cells, particularly osteoprogenitors and osteoblasts.

Angio-osteogenic interactions are particularly evident during early bone formation. Hypertrophic chondrocytes in primary ossification centers promote vascular invasion through the secretion of pro-angiogenic factors, including vascular endothelial growth factor (VEGF) and fibroblast growth factors (FGFs) [30, 34–36]. Type H capillaries provide nutrients and secrete platelet-derived growth factor (PDGF), noggin, FGFs, and transforming growth factors (TGF) to support osteoprogenitor maintenance and self-renewal [13]. In turn, osteoprogenitors regulate vascular phenotype through the expression of Slit guidance ligand 3 (SLIT3) and VEGF [37–39]. The Notch pathway is a central mediator of this coupling: activation of endothelial Notch signalling, notably through Delta-like ligand 4 (DLL4), induces noggin production, promoting osteoprogenitor differentiation, endothelial proliferation, and vessel formation in postnatal long bones [20, 40–42]. Accordingly, inhibition of DLL4/Notch signalling suppresses type H angiogenesis, leading to impaired osteogenesis [43]. Bone morphogenic proteins (BMPs) further contribute by stimulating both osteogenesis and angiogenesis [14].

The role of the vasculature is equally important during bone repair [44, 45]. Beyond ensuring oxygen and nutrient supply, blood vessels allow the recruitment of osteoprogenitors to the fracture environment [46]. Impaired blood flow delays bone healing, whereas VEGF-A produced by osteoblastic lineage cells promotes macrophage recruitment, angiogenesis, vascular growth, osteoclast recruitment, and cartilage resorption throughout the repair process [47, 48]. Additional factors, including FGFs, PDGF, and BMPs, also contribute to coordinating angiogenesis and bone regeneration [14].

These findings highlight the central role of blood vessel-bone crosstalk in osteogenesis and repair. While interactions between blood ECs and osteogenic cells are well established, they represent only one component of the vascular network regulating skeletal homeostasis. Recent studies identifying lymphatic vessels in bone challenge the traditional view that the skeleton lacks a lymphatic network and suggest that lymphatic vessels also contribute to bone homeostasis and repair.

## Bone Lymphatics Vasculature: Structure and Emerging Functions

The lymphatic system regulates interstitial fluid homeostasis, waste clearance, and immune surveillance through a network of initial and collecting lymphatic vessels that transport interstitial fluid from tissues to lymph nodes and back to the blood circulation [49–51]. Initial lymphatic capillaries, the primary sites of lymph formation, are composed of lymphatic ECs. They are characterized by discontinuous “button-like” junctions, a discontinuous basement membrane, blind-ended architecture, and overlapping endothelial cells forming primary valves, features that facilitate the uptake of interstitial fluid, macromolecules, and immune cells while preventing reflux [52]. Initial lymphatics converge into larger collecting vessels surrounded by a continuous basement membrane and lymphatic muscle cells that generate contractile forces to propel lymph. These vessels contain intraluminal secondary valves that ensure unidirectional lymph flow [52].

The presence of lymphatic vessels in bone has only recently been recognized, owing to advances in tissue-clearing techniques, light-sheet microscopy, and the use of lymphatic endothelial markers such as lymphatic vascular endothelial hyaluronan receptor 1 (LYVE-1), prospero homeobox protein 1 (PROX1), and podoplanin (PDPN) [53–55]. These vessels display the typical morphology of lymphatic capillaries and appear predominantly localized within the periosteum and medullary regions, although their anatomical organization and drainage routes remain incompletely defined [56, 57].

Beyond their classical drainage and immune functions, lymphatic vessels have emerged as important regulators of bone repair. Lymphatic ECs promote skeletal and hematopoietic regeneration through the secretion of lymphangiocrine factors, including VEGF-C, IL-6 (interleukin-6 ), and C-X-C Motif Chemokine Ligand 12 (CXCL12), which support lymphangiogenesis, angiogenesis, hematopoietic stem cell maintenance, and bone formation following injury [57]. Enhanced lymphatic drainage after fracture is also associated with reduced inflammation, increased M2 macrophage polarization, improved osteoblast survival, and more efficient healing. Together, these findings identify the bone lymphatic network as an important component of the skeletal microenvironment and a potential therapeutic target in bone diseases and impaired healing [50].

Overall, these observations highlight the importance of both blood and lymphatic vessels in maintaining skeletal homeostasis and supporting bone repair. However, vascular networks do not act independently within the bone microenvironment. Emerging evidence suggests that the bone vasculature is also functionally connected to the osteocyte network embedded within the mineralized matrix. As the most abundant cells in bone and the principal mechanosensors of the skeleton, osteocytes are ideally positioned to coordinate communication between the vascular and skeletal compartments.

## Osteocytes

Within bone, osteocytes influence nearly all cell types involved in skeletal homeostasis through multiple modes of communication. These include direct cell-cell interactions as well as paracrine signalling mediated by the secretion of various factors [58]. Together, these communication pathways regulate key bone processes, such as remodelling and repair, which are discussed in the following sections of this review.

## Osteocyte Regulation of Bone Remodelling

Through their extensive lacuno-canalicular network, osteocytes coordinate osteoblast and osteoclast activity and are central regulators of bone remodelling [59]. In response to mechanical loading, they regulate bone formation and resorption through key pathways including RANKL/OPG and SOST/DKK1/Wnt. Osteocytes promote osteoclastogenesis through receptor activator of nuclear factor-κB ligand (RANKL) and macrophage colony-stimulating factor (M-CSF), while osteoprotegerin (OPG) and nitric oxide (NO) inhibit osteoclast activity [60–64]. Bone formation is stimulated by NO and adenosine triphosphate (ATP), while sclerostin (SOST), Dickkopf-related protein 1 (DKK1), suppress Wnt-mediated osteoblast activity [65–67]. In addition, neuropeptide Y (NPY), highly expressed by osteocytes, has emerged as an inhibitor of osteoblast activity [68]. Through these mechanisms, osteocytes maintain the balance between bone resorption and formation required for skeletal homeostasis.

### Osteocyte Regulation of Bone Repair

Recent studies suggest that osteocytes contribute to stress fracture repair through changes in network morphology and molecular expression within the lesion microenvironment [8]. Choy et al., described the chronological sequence of cellular events regulated by osteocytes during the regeneration of non-critical bone lesions (microfractures) [8].

During the early phase, osteocytes adjacent to the fracture undergo apoptosis and express caspase-3, while neighbouring viable osteocytes produce IL-6 and cyclooxygenase-2 (COX-2), initiating inflammation. Apoptotic osteocytes act as alarm signals, inducing the expression of RANKL, OPG, and VEGF in surrounding osteocytes to promote osteoclast recruitment and angiogenesis [5, 6]. Histological analyses of fatigue fractures further revealed increased IL-6 and COX-2 and reduced SOST expression near the lesion [6, 7].

During the intermediate phase, osteocyte markers E11/Podoplanin and dentin matrix protein-1 (DMP-1) are upregulated while SOST expression remains reduced, promoting osteogenesis and supporting maintenance of the lacuno-canalicular network through increased E11/Podoplanin and connexin-43 expression [7, 8, 69, 70].

In the late phase, bone healing progresses with callus remodelling and mineralization, and restoration of the lacuno-canalicular network is associated with reduced DMP-1, E11/Podoplanin, and connexin-43 expression, increased sclerostin levels, and elevated matrix extracellular phosphoglycoprotein (MEPE), which may contribute to callus mineralization [8]. Together, these findings indicate that osteocytes exhibit both temporal and spatial regulation of their molecular profile during bone repair.

## Mechanisms of Osteocyte-blood Vessel Communication

**Fig. 1:**
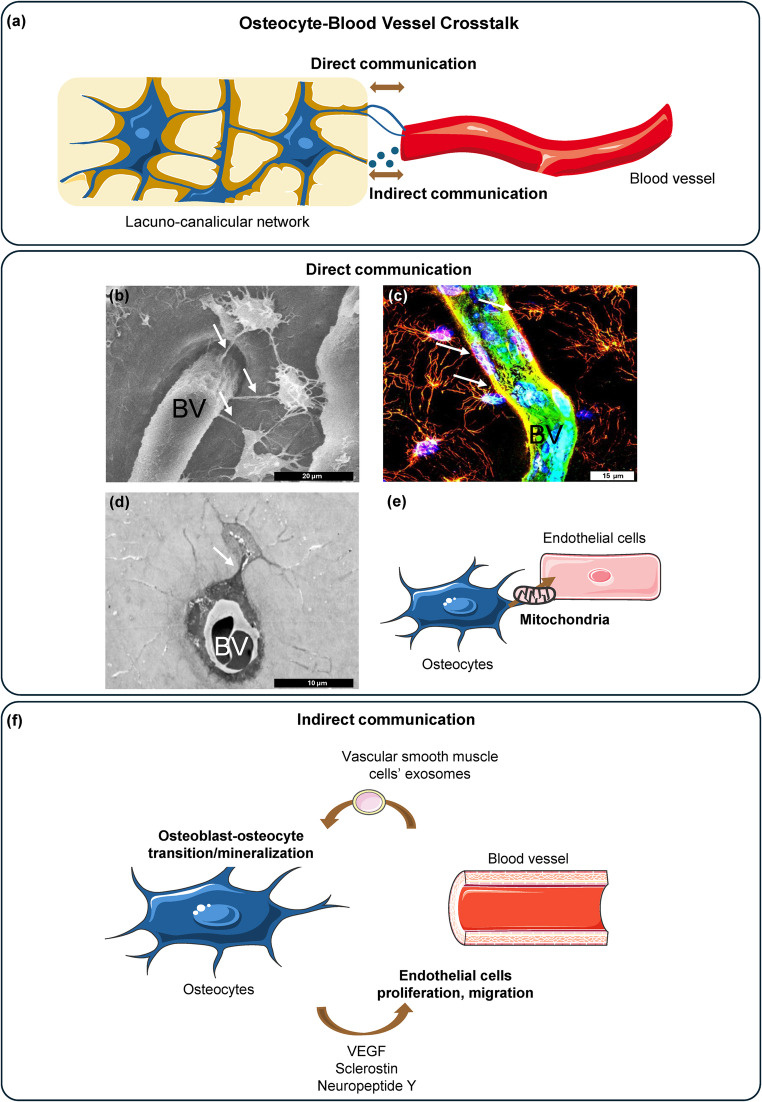
Osteocyte-blood vessel crosstalk. (**a**) Communication between osteocytes and blood vessels occurs through indirect/paracrine and direct communication. Physical interactions between blood vessels and osteocytes were observed in different studies, suggesting direct communication. (**b**) Scanning electron microscopy of sheep bone (3 y/o) after a sinus lift procedure from *Fricain et al.*. (image not published), imaged with a scanning electron microscope at 20 kV, using the 2000× magnification [20]. BV indicates blood vessels, and white arrows indicate dendrites of osteocytes in contact with the blood vessels. (**c**) Confocal image showing phalloidin-stained (red), decalcified calvaria from a 12-week-old Flk1-GFP mouse, produced by our team (maximum intensity z-projection, z size of 20 μm with z step size of 0,40 μm, 63x oil objective). BV indicates GFP^+^ blood vessels, and white arrows indicate dendrites of osteocytes in contact with the blood vessels. (**d**) Transmission electron microscopy of tibia from adult male Wistar rats. BV indicates blood vessels, and white arrows indicate dendrites of osteocytes in contact with the blood vessels. (**e**) Direct communication is required to maintain a normal transcortical vessel (TCV) network, in part via osteocytes’ mitochondrial transfer to ECs. (**f**) Indirect signalling involves paracrine factors secreted by osteocytes, such as VEGF, sclerostin, and neuropeptide Y, which modulate ECs’ behaviour. Extracellular vesicles, like exosomes, released by vascular cells, regulate the transition of osteoblasts through osteocytes, promote osteocyte-like characteristics and mineralization.

The regulatory role of osteocytes in bone remodelling and repair should be considered within the context of a highly vascularized tissue. However, to date, the relationship between osteocytes and bone vascularisation remains poorly understood. The physical interactions between osteocyte dendrites and blood vessels, and the paracrine activity of osteocytes, suggest the existence of both direct and indirect modes of communication between osteocytes and the bone vascular network (Fig. 1a).

## Direct Communication

Several studies have demonstrated in vivo a physical interaction between osteocyte dendrites and blood vessels, suggesting that osteocytes may communicate with ECs through their canaliculi [17–21] (Fig. 1b, c and d).

Beyond the maintenance of cellular homeostasis, intercellular mitochondrial transfer has recently emerged as a mechanism of cell-to-cell communication. TCVs are a key component of the bone vascular network, but the mechanisms regulating their formation and maintenance are still not fully understood. Liao et al. showed that osteocytes are required to maintain a normal TCV network, partly via mitochondrial transfer to ECs [18]. Using heterozygous Dmp1^Cre^-DTA^ki/wt^ mice, which allow partial osteocyte ablation via the diphtheria toxin, they observed TCV regression and reduced expression of angiogenic genes. After demonstrating in vitro that mitochondria can be transferred from osteocytes to EC, the researchers used another transgenic mouse line, Dmp1^Cre^-Cox8^Dendra2^, in which osteocyte mitochondria are specifically labelled with the green fluorescent protein Dendra2. Using confocal microscopy, the authors revealed colocalization of Dendra2-labeled osteocyte mitochondria with CD31-labeled ECs, confirming mitochondrial transfer in vivo [18]. Moreover, the authors showed that transferred mitochondria promote angiogenesis by stimulating D-sphingosine production in ECs. These findings raise the possibility that organelle transfer to ECs represents an emerging mode of communication for osteocytes, potentially contributing to their interaction with the vascular compartment (Fig. 1e) [18].

In bone, intercellular communication is largely mediated by gap junctions composed of connexins, which allow the diffusion of molecules smaller than 1 kDa. Within the osteocyte network, this communication primarily relies on connexin 43, allowing the transfer of ions and small signalling molecules between cells [71, 72]. Beyond its role in mediating the release of factors in response to mechanical stimuli, connexin 43-dependent signalling also promotes osteocyte survival and preserves the integrity of the lacuno-canalicular network under mechanical stress. Given the reported physical interactions between osteocytes and blood vessels, it is possible that gap junctions may contribute to this communication. Although their role in direct osteocyte-vascular interactions has not yet been investigated, they may represent a potential mechanism underlying osteocyte-vascular coupling.

Investigating the direct communication in vivo remains technically challenging because osteocytes and blood vessels are embedded within the mineralized bone matrix. Consequently, research has mainly been focused on paracrine interactions between osteocytes and vascular cells.

### Indirect/Paracrine Communication

Beyond direct physical interactions, osteocytes and vascular cells are involved in dynamic paracrine communication through the secretion of different proteins and extracellular vesicles (EVs) (Fig. 1f).

 In vitro studies demonstrate a communication via the secretion of several molecules between osteocytes and ECs. Conditioned media from Murine Long-bone Osteocyte Y4 (MLOY4) stimulate Human Umbilical Vein ECs (HUVEC) migration, tubule formation, and upregulate angiogenic genes (VEGF, VEGF-Receptor 2, PDGF-Receptor-α, Angiopoietin-1, von Willebrand factor, matrix metalloproteinase-9) [22]. Osteocyte-derived VEGF activates the VEGFR2-MAPK/ERK pathway in ECs, and its inhibition reduces osteocyte-mediated angiogenesis [22]. These findings indicate that, like osteoblasts, osteocytes are a key VEGF source and contribute to bone angiogenesis regulation [22, 73]. Then, a study identified a new angiogenic role of sclerostin, suggesting it as a potential mediator of angiogenesis-osteogenesis coupling [23]. In vitro, sclerostin increased the proliferation of HUVEC and promoted anastomosed tubular network formation, enhancing tube number, total length, and junctions. Chen et al., showed recently that the osteocyte-derived NPY inhibits bone angiogenesis by reducing preosteoclast proliferation and platelet-derived growth factor-BB (PDGF-BB) secretion [24]. By decreasing PDGF-BB production, NPY suppresses ECs migration and tubular network formation via PI3K/Akt pathway inhibition [24]. In vivo, in ovariectomized mice, osteocyte-derived NPY significantly impairs the formation of type H vessels and aggravates bone loss. Together, these findings highlight that osteocytes actively regulate bone angiogenesis through a complex paracrine network involving both pro- and anti-angiogenic factors.

Osteocytes are embedded within the mineralized bone matrix, suggesting that they normally reside in a relatively low-oxygen environment. However, the exact oxygen concentration inside osteocytes’ lacunae remains difficult to determine due to technical limitations. Moreover, hypoxia can occur in pathological contexts, such as bone fractures, where vascular disruption leads to localized oxygen deficiency [74]. Prolonged hypoxia has been associated with osteocyte apoptosis and subsequent osteoclast recruitment from bone marrow precursors [75]. Conversely, hypoxia can also promote bone formation through activation of the Wnt/β-catenin pathway, as illustrated by osteocyte-specific deletion of *Vhl* or *Phd2*, which reduces *Sost* expression and leads to a high bone mass phenotype in mice [76, 77]. Together, these findings suggest that hypoxia, via HIF-1α signalling, may modulate osteocyte function and influence their communication with ECs. Our team investigated the paracrine interaction between osteocytes and ECs by analysing the effect of conditioned media from MLO-Y4 cells, grown under normoxia (21% O_2_) or hypoxia (5% and 1% O_2_), on endothelial gene expression (*data not published*). To assess changes in gene expression in ECs, RNA sequencing was performed. Our results indicate that osteocyte-derived factors secreted in hypoxia did not significantly alter gene expression in EC, suggesting that under these conditions, paracrine signalling may not be the dominant mode of communication involved. Further studies are required to clarify the mechanisms by which oxygen levels shape osteocyte physiology.

Osteocyte communication extends beyond classical paracrine signalling by soluble factors. In recent years, extracellular vesicles (EVs) have emerged as a novel mode of intercellular communication, notably within the bone microenvironment. These vesicles play a key role in paracrine signalling through the proteins, lipids, and nucleic acids they carry, which exert specific biological functions [78]. Increasing evidence indicates that EVs contribute to the crosstalk between bone cells and the vasculature [79]. For example, EC-derived EVs participate in angiogenesis-osteogenesis coupling during bone healing, carrying factors such as VEGF, angiopoietin-1, and FGF2, which promote EC proliferation, migration, and tube formation [79]. Exosomes are small EVs (≈ 30–150 nm) formed within the endosomal compartment and released by exocytosis. A recent study by Fernandes et al., showed that the exosomes of vascular smooth muscle cells regulate the transition of osteoblasts through osteocytes [25]. These exosomes carry transcripts associated with the osteocytic phenotype and components of the WNT/β-catenin pathway, promoting osteocyte-like characteristics and mineralization through high ATP levels and induction of mineralization-related genes. However, exosomes derived from ECs promote only the differentiation of osteoblasts via TGF-β1, Osterix, and RUNX2 [25]. These findings highlight the potential key role of vascular-derived exosomes in the vascular-bone cellular communication. Emerging evidence suggests that osteocyte-derived EVs contribute to bone tissue regulation [80, 81]. Indeed, a recent study showed that osteocyte-derived EVs can enhance osteoblast differentiation notably through tropomyosin-1-mediated effects on matrix stiffness [81]. In contrast, their role in osteocyte-vascular communication remains poorly defined, as there is currently no evidence addressing whether osteocyte-derived EVs can influence ECs’ behaviour or participate in the regulation of bone vascularisation.

Altogether, these findings indicate that osteocytes actively regulate bone vascularisation through multiple modes of communication, including direct cellular interactions with mitochondrial transfer and paracrine signalling (Fig. 1**)**. However, the precise mechanisms governing this crosstalk remain incompletely understood. To date, most evidence supports a predominantly unidirectional influence of osteocytes on the vascular compartment, except for extracellular vesicle-mediated communication. In addition, the development of advanced experimental tools in vivo may help reveal additional modes of communication between osteocytes and blood vessels.

### Potential Osteocyte-lymphatics Interaction

Direct interactions between osteocytes and lymphatic vessels remain largely unexplored. Nevertheless, several observations suggest the existence of a potential functional crosstalk [49]. Osteocytes are highly dependent on fluid flow within the lacuno-canalicular network, a key regulator of mechanotransduction and cell function. Current evidence suggests that bone interstitial fluid may ultimately drain toward periosteal lymphatic vessels through pathways involving the Haversian and Volkmann canals, although the precise drainage routes remain incompletely defined [82]. By regulating the clearance of interstitial fluid, inflammatory mediators, and bone remodelling, lymphatic vessels may contribute to maintaining the osteocyte pericellular environment. Conversely, osteocytes secrete numerous angiogenic and immunomodulatory factors that could potentially influence lymphatic ECs’ function. Although these hypotheses remain to be experimentally validated, osteocyte-lymphatic communication was identified as a promising and largely unexplored area of skeletal biology.

### Osteocyte-crosstalk in Bone Disease and Aging

Osteocyte-vascular communications are implicated in several pathological contexts and aging (Table 1). Understanding these interactions better may therefore provide new insights into the mechanisms underlying skeletal disorders and identify potential therapeutic targets.

### Bone Repair

Vascularisation is essential for bone repair, and osteocyte-stimulated angiogenesis may occur during the early and intermediate stages of regeneration [8]. As described in the section “Osteocyte Regulation of Bone Repair”, studies suggest that osteocytes differentially express molecules in the environment of a microfracture. Cheung et al. showed, in vitro, that apoptotic osteocytes release pro-angiogenic factors, including VEGF, promoting ECs proliferation, migration, and tubule formation [83]. VEGF inhibition abolishes these effects. Moreover, growth factors and BMPs secreted by osteocytes during the early phase of bone regeneration are necessary for the revascularisation and neo-angiogenesis of the bone callus [84]. Hadjiargyrou et al., in a rat model of femur fracture with an intramedullary spin, identified that immature osteocytes expressed cysteine-rich angiogenic inducer 61 (CYR61) in the trabecular bone of the callus at day 10 and day 21 post-fracture. CYR61, involved in inflammation and angiogenesis, is no longer expressed once osteocytes fully mature [85]. Then, as mentioned above, the communication between osteocytes and ECs via direct mitochondria transfer promotes angiogenesis by stimulating the production of D-sphingosine in EC [18]. Moreover, Liao et al. treated mice with femoral bone defects with oral D-sphingosine for seven days, resulting in increased vascular branching, accelerated TCV formation, and enhanced bone regeneration [18]. The interaction between osteocytes and bone vasculature emerges during the early and intermediate stages of bone regeneration. A deeper understanding of these interactions will provide new insights into osteocyte-blood vessel crosstalk and may inspire potential application therapies for bone healing.

### Osteonecrosis

Osteonecrosis is a disease most frequently affecting the hip joint, particularly following trauma. However, the administration of glucocorticoids constitutes the main cause of non-traumatic osteonecrosis [86–88]. Osteonecrosis of the femoral head (ONFH) is an irreversible and progressive disorder of the hip that, over time, progresses toward dysfunction and collapse of the femoral head, leading to disability. Interactions between osteocytes and the vascular network appear to be involved in the pathological process of ONFH associated with glucocorticoids.

Osteonecrosis induced by glucocorticoids leads to the apoptosis of osteocytes [89, 90]. Numerous apoptotic osteocytes and empty lacunae were observed in femoral heads from total hip arthroplasties [91]. Increased osteocyte apoptosis is associated with reduced VEGF, impaired bone angiogenesis, and decreased bone strength [92].

A recent study shows the involvement of sclerostin in the pathological process of ONFH associated with glucocorticoids [93]. *In vivo* analyses revealed a decrease in alkaline phosphatase and VEGF staining in necrotic areas, and increased sclerostin deposition versus healthy tissue. Dexamethasone, commonly used to establish experimental models of ONFH, stimulates osteocytes to secrete sclerostin, thereby enhancing its inhibitory effects on osteogenesis and angiogenesis. Furthermore, *in vitro*, ECs and osteocyte co-culture enhanced dexamethasone’s inhibitory effects on EC proliferation and tubulogenesis compared with EC monoculture. The authors show that osteocyte-derived sclerostin impairs these two processes by inhibiting the Wnt pathway [93]. The cellular mechanisms involved in osteonecrosis also involve other cell types that are not described in this review [90].

### Aging

Both osteocytic and vascular networks undergo significant alterations during aging. In DBA/1DEREG mice, aging is associated with a reduction in transcortical vessels (TCVs) [29]. Tiede-Lewis et al. described a decline in osteocyte density together with a reduction in the number of dendritic processes per osteocyte in both male and female mice [94]. Palmier et al. further demonstrated that aging concurrently affects the vascular and osteocytic networks, leading to decreases in vessel density and osteocyte dendricity in cortical bone [21]. Although no direct causal relationship was identified, these findings suggest that both networks deteriorate in parallel during aging.

Beyond the structural deterioration of the osteocyte network, age-related loss of dendritic processes and lacuno-canalicular connectivity has been proposed to impair osteocyte mechanosensitivity and reduce the ability of osteocytes to coordinate adaptive bone remodelling in response to mechanical loading [95]. Aging is also associated with the accumulation of senescent osteocytes [96]. These cells acquire a senescence-associated secretory phenotype (SASP), characterized by the increased production of pro-inflammatory cytokines (IL-6, IL-1α, GATA4) and osteoclastogenic factors. Consistent with this, Kim et al. demonstrated that senescence-induced RANKL expression in osteocytes contributes to age-related cortical bone loss, highlighting the functional impact of osteocyte senescence on skeletal homeostasis [97]. These changes may compromise the ability of osteocytes to regulate bone remodelling and could potentially alter their interactions with the vascular microenvironment. Although the direct consequences of osteocyte senescence on the bone vasculature remain poorly understood, age-related changes in the osteocyte secretome could influence neighboring ECs and thereby contribute to alterations in the bone vascular niche.

In parallel, age-related vascular dysfunction is characterized not only by a reduction in TCV abundance but also by EC dysfunction and decreased angiocrine signalling, which contribute to impaired bone regeneration and skeletal homeostasis [98]. The age-associated decline in osteogenic type H vessels, a specialized endothelial subtype that couples angiogenesis and osteogenesis, has been linked to reduced bone formation and impaired regenerative capacity [13, 38]. While Palmier et al. found that osteocyte intracellular oxygen levels were not directly related to local vascular density in cortical bone [21], these findings do not exclude functional interactions between osteocytes and the vascular network. Indeed, vascular aging may influence osteocyte function through mechanisms extending beyond oxygen availability, including alterations in nutrient supply and EC dysfunction. Conversely, osteocytes contribute to the regulation of bone vascularization through the production of factors such as VEGF, suggesting that age-related alterations in osteocyte viability, mechanosensitivity, and secretory activity may further impact vascular homeostasis.

Recent studies have also identified functional intraosseous lymphatic vessels involved in bone regeneration and skeletal homeostasis. While aging has been shown to impair lymphatic EC function and lymphangiogenic signalling, the potential interactions between osteocytes and the lymphatic network during skeletal aging remain largely unexplored [98].

Together, these observations support the concept that aging induces a progressive deterioration of both osteocytic and vascular networks, potentially disrupting osteocyte-vascular communication and contributing to the decline in skeletal integrity observed in aged bone.

**Table 1:** Involvement of osteocyte-vascular crosstalk in bone diseases and aging

Context	Signalling modality	Key mediator(s)	Functional consequences
Bone repair	Paracrine, soluble factors	VEGF, BMPs, CYR61 (secreted by osteocytes)	Increased ECs proliferation, migration, and tube formation; revascularisation of the bone callus [83–85]
	Organelle transfer	Transfer of mitochondria from osteocytes to ECs; D-sphingosine	Formation of TCVs; enhanced bone regeneration [18]
Osteonecrosis	Paracrine, soluble factors	Decreased VEGF and increased sclerostin secretion by osteocytes; inhibition of Wnt signalling	Reduced blood vasculature; impaired bone formation [93]
Aging		Unknown	Decreased osteocyte number and dendrite density; reduced TCVs; impaired lymphatic EC function; the relationship between osteocytes and vascularisation remains unclear [21, 98]

*BMP* Bone morphogenetic, *CYR61* cysteine-rich angiogenic inducer 61, *EC* Endothelial cell, *TCV* transcortical vessel, *VEGF* Vascular endothelium growth factor

### Conclusions and Future Directions

Over the past two decades, scientific advances have demonstrated that osteocytes are dynamic cells that regulate bone cells’ activity and influence bone homeostasis. In parallel, bone vascularisation plays an essential role in maintaining this homeostasis by contributing to bone formation, maintenance, and repair. However, the interactions between osteocytes and blood vessels, and notably ECs, remain poorly studied. Available data suggest that this communication occurs through several modalities. It may be indirect, via paracrine mechanisms in which osteocytes secrete molecules that modulate EC behaviour, or direct, through physical interactions such as mitochondrial transfer and potentially gap junctions. These interactions appear to contribute to bone repair and may also be involved in certain bone pathologies.

Despite these advances, many questions remain unanswered regarding the cellular and molecular mechanisms involved in these interactions. Most studies have focused on the influence of osteocytes on ECs, whereas the reciprocal effects of ECs and their secreted factors on osteocyte biology and function remain largely unexplored. In addition, the recent identification of lymphatic vessels in bone has opened a new area of investigation. Although potential interactions between osteocytes and the lymphatic vasculature have been proposed, their nature, molecular mediators, and functional significance remain largely unknown.

The study of osteocyte biology is challenging due to limited access to mineral-embedded osteocytes and the absence of fully representative *in vitro* models. Developing new tools would allow a better understanding of the mechanisms involved. For example, laser microdissection seems interesting to isolate osteocytes, particularly those in contact with vessels, and to analyse the expression of their genes [99]. Omics approaches, such as spatial transcriptomics and proteomics, could allow better characterization of the molecular modifications affecting osteocytes and thus open new research directions. The improvement of intravital imaging techniques allows the study of fundamental osteocytic functions at the subcellular scale *in vivo* while preserving local and systemic signals [100]. These approaches could constitute relevant tools to analyse the modes of direct and indirect communication between osteocytes and blood vessels.

Elucidating interactions between osteocytes and bone vasculature may provide new insight into bone physiology, disease mechanisms, and help identify new therapeutic strategies.

## Data Availability

No datasets were generated or analysed during the current study.
